# Neurodevelopmental Outcome and Neuroimaging of Very Low Birth Weight Infants from an Italian NICU Adopting the Family-Centered Care Model

**DOI:** 10.3390/children11010012

**Published:** 2023-12-21

**Authors:** Licia Lugli, Marisa Pugliese, Natascia Bertoncelli, Luca Bedetti, Cristina Agnini, Isotta Guidotti, Maria Federica Roversi, Elisa Muttini Della Casa, Francesca Cavalleri, Alessandra Todeschini, Antonella Di Caprio, Tommaso Zini, Lucia Corso, Francesca Miselli, Fabrizio Ferrari, Alberto Berardi

**Affiliations:** 1 Neonatology Unit, Mother-Child Department, University Hospital of Modena, Via del Pozzo 71, 41100 Modena, Italy; natascia.bertoncelli@aou.mo.it (N.B.); bedetti.luca@aou.mo.it (L.B.); cristina.agnini@studenti.unipd.it (C.A.); guidotti.isotta@aou.mo.it (I.G.); roversi.federica@aou.mo.it (M.F.R.); dellacasa.elisa@aou.mo.it (E.M.D.C.); 163320@studenti.unimore.it (T.Z.); francesca.miselli@unimore.it (F.M.); fabrizio.ferrari@unimore.it (F.F.); alberto.berardi@unimore.it (A.B.); 2 Psychology Unit, University Hospital of Modena, 41100 Modena, Italy; marisa.pugliese@unimore.it; 3 Neuroradiology Unit, University Hospital of Modena, 41100 Modena, Italy; cavalleri.francesca@aou.mo.it (F.C.); todeschini.alessandra@aou.mo.it (A.T.); 4 Department of Medical and Surgical Sciences for Mother, Children and Adults, Postgraduate School of Pediatrics, University of Modena and Reggio Emilia, 41121 Modena, Italy; 261005@studenti.unimore.it (A.D.C.); 196397@studenti.unimore.it (L.C.); 5 PhD Program in Clinical and Experimental Medicine, University of Modena and Reggio Emilia, 41121 Modena, Italy

**Keywords:** preterm infants, very low birth weight, neurodevelopmental outcomes, brain magnetic resonance imaging

## Abstract

Background: Improvements in perinatal care have substantially decreased mortality rates among preterm infants, yet their neurodevelopmental outcomes and quality of life persist as a pertinent public health concern. Family-centered care has emerged as a holistic philosophy that promotes effective alliances among patients, families, and healthcare providers to improve the quality of care. Aims: This longitudinal prospective study aims to evaluate the neurodevelopmental outcomes and brain MRI findings in a cohort of preterm newborns admitted to a neonatal intensive care unit (NICU) adopting a family-centered care model. Methods: Very low birth weight (VLBW) infants admitted to the NICU of Modena between 2015 and 2020 were enrolled. Infants who underwent conventional brain magnetic resonance imaging (MRI) at term-equivalent age were included. Neurodevelopmental follow-up was performed until the age of 24 months by a multidisciplinary team using the Amiel-Tison neurological assessment and the Griffiths Mental Developmental Scales (GMDS-R). Neurodevelopmental outcomes were classified as major sequelae (cerebral palsy, DQ ≤ 70, severe sensory impairment), minor sequelae (minor neurological signs such as clumsiness or DQ between 71 and 85), and normal outcomes (no neurological signs and DQ > 85). Risk factors for severe outcomes were assessed. Results: In total, 49 of the 356 infants (13.8%) died before hospital discharge, and 2 were excluded because of congenital disorders. Of the remaining 305 infants, 222 (72.8%) completed the 24 month follow-up and were included in the study. Neurodevelopmental outcomes were classified as normal (*n* = 173, 77.9%), minor (*n* = 34, 15.3%), and major sequelae (*n* = 15, 6.8%). Among 221 infants undergoing brain MRI, 76 (34.4%) had major lesions (intraventricular hemorrhage, hemorrhagic parenchymal infarction, periventricular leukomalacia, and large cerebellar hemorrhage). In the multivariate regression model, the retinopathy of prematurity (OR 1.8; *p* value 0.016) and periventricular–intraventricular hemorrhage (OR 5.6; *p* value < 0.004) were associated with major sequelae. Conclusions: We reported low rates of severe neurodevelopmental outcomes in VLBW infants born in an Italian NICU with FCC. Identifying the risk factors for severe outcomes can assist in tailoring and optimizing early interventions on an individual basis, both within the NICU and after discharge.

## 1. Introduction

Preterm delivery contributes significantly to neonatal and pediatric mortality and morbidity worldwide [1,2]. Modern neonatal intensive care methods have played a key role in improving the survival rates of neonates born at the threshold of viability [3,4,5,6,7,8,9]. However, approximately 5–10% of all very low birth weight (VLBW, birth weight < 1500 g) infants still exhibit significant motor impairments, such as cerebral palsy (CP), and up to 25–50% display cognitive, behavioral, and/or attention impairments. These neurodevelopmental disabilities can have a profound impact not only on affected individuals and their families but also on public healthcare resources, especially due to the lifelong nature of the sequelae [1,3]. The third trimester of gestation is a period of intense growth and development of the fetal central nervous system. Preterm birth disrupts this delicate process and forces fetal development to continue in the potentially hostile extrauterine environment of the NICU [3]. Parents and professional caregivers can work together to minimize the negative impact of the NICU experience, hopefully reducing subsequent neurodevelopmental impairment and disability. Based on sensory input from various stimuli, the brain can undergo both temporary and permanent changes, strengthening or creating new neuronal synaptic connections. The most effective neuroprotective intervention is parental care [3,10,11], which cannot be provided by professionals alone. The family is the single “constant” in the infant’s life, as it provides a unique emotional and nurturing connection that lasts over time [10,11,12,13]. To acknowledge the growing recognition of the challenges faced by parents in the NICU setting, numerous neonatal intensive care units (NICUs) globally have embraced the tenets of family-centered care (FCC) as an integral part of their caregiving protocols. FCC has emerged as a holistic philosophy that promotes effective alliances among patients, families, and healthcare providers to improve the quality of care. FCC is a care approach based on mutual collaboration between healthcare professionals and parents, emphasizing the utmost respect for individual dignity, active participation, and the sharing of responsibilities. Parents are the most important nurturers of the infant, and the infant becomes a guide for care. FCC facilitates a transition from conventional, task-oriented care to a relational approach. NICU professionals are encouraged to acknowledge the developmental needs of preterm infants, provide support to parents in nurturing their relationship with their newborn, and foster the enhancement of parental competencies [11,12]. The points mentioned above emphasize the increasing importance of assessing the neurodevelopmental outcomes of premature infants admitted to the NICU. Brain magnetic resonance imaging (MRI) is increasingly used in the preterm population to complement cranial ultrasound (CUS) to improve prognostic information and guide current clinical care and future supportive care. Furthermore, information obtained from MRI findings can reduce parental anxiety about their preterm infant [14,15,16,17,18,19]. MRI has emerged as a secure and effective technique for evaluating preterm brain development, with numerous studies outlining typical acquired injuries. Advanced MRI techniques further highlight widespread developmental alterations associated with premature birth. [3,13,14,15,16,17,18,19]. Despite the notable advancements in our understanding of preterm brain injury facilitated by MRI, the precise prediction of individual neurodevelopmental outcomes remains challenging. Prior studies have predominantly reported findings at the group level, presenting individual outcomes in small cohorts or emphasizing group differences in larger cohorts. Additionally, discrepancies arise due to variable classifications of lesions, and in many cases, brain MRI is performed in response to abnormalities found on CUS [13,14,15,16,17,18,19]. This longitudinal prospective study aims to evaluate neurodevelopmental outcomes and brain MRI findings in a cohort of preterm newborns from a NICU adopting the FCC model. In this study, we document the rate and spectrum of lesions found in a cohort of preterm infants undergoing routine imaging at term equivalent age (TEA) and relate these findings to neurodevelopmental outcomes.

## 2. Aims of the Study

This study aims to conduct the following:

(1) Describe neurodevelopmental outcomes at 24 months of corrected age (CA) in a cohort of VLBW infants admitted to a single Italian NICU adopting FCC.
(2) Identify perinatal risk factors for severe neurodevelopmental outcomes.
(3) Evaluate the correlation between brain MRI findings at TEA and neurodevelopmental outcomes.

## 3. Materials and Methods

### 3.1. Study Design

VLBW infants admitted to the NICU of Modena, Italy, between 2015 and 2020 were enrolled in this study. The exclusion criteria were major congenital malformations and the diagnosis of a genetic disorder. The Ethics Committee of the University Hospital of Modena and Reggio Emilia granted approval for this study (protocol 205/2015, no. 4818). Written consent was obtained from the parents of each participating infant. Various perinatal factors were assessed, including birth weight (BW), gestational age (GA), delivery site and method, ethnicity, gender, multiple gestations, prenatal steroid exposure, Apgar scores at the first and fifth minute, sepsis, mechanical ventilation, ultrasound-detected cerebral lesions (intraventricular–periventricular hemorrhage, periventricular leukomalacia), patent ductus arteriosus (PDA) treatment, necrotizing enterocolitis (NEC), retinopathy of prematurity (ROP), and breastfeeding at discharge. ROP was classified according to the International Classification for Acute Retinopathy of Prematurity based on its severity [20]: stage 0, immature retinal vasculature with no ROP; stage 1, demarcation line between vascularized and avascular retina; stage 2, ridge (demarcation line with height, width, and volume) +/− small tufts of neovascular tissue; stage 3, ridge with extraretinal fibrovascular proliferation; stage 4, partial retinal detachment (4A: extrafoveal detachment; 4B: retinal detachment including fovea); stage 5, total retinal detachment. Moreover, plus disease was defined as the vascular dilatation (venous) and tortuosity (arteriolar) of posterior retinal vessels in at least 2 quadrants of the retina. IVH, detected by CUS, was classified as follows: grade I, hemorrhage limited to the germinal matrix or occupying ≤10% of the ventricle; grade II, IVH occupying >10% and ≤50% of the ventricle without ventricular enlargement; grade III, IVH occupying > 50% of the ventricle; grade IV, IVH with intraparenchymal hemorrhagic infarction (PIH). Grades III and IV are referred to as “severe IVH” [14,21,22,23]. PVL, diagnosed by CUS, was classified as follows: grade 1, areas of increased periventricular echogenicity without any cyst formation persisting for more than 7 days; grade 2, the echogenicity has resolved into small periventricular cysts; grade 3, areas of increased periventricular echogenicity that develop into extensive periventricular cysts in the occipital and frontoparietal region; grade 4, areas of increased periventricular echogenicity in the deep white matter developing into extensive subcortical cysts [14,24]. Bronchodysplasia was defined as the need for oxygen supplementation at 36 weeks of postconceptional age [25].

### 3.2. Family-Centered Care

Since 2002, the NICU of Modena has adopted the FCC model for the care of newborns and their parents. The NICU provides round-the-clock access, seven days a week, to parents [12]. FCC involves the family as an essential contributor to the provision of individualized, developmentally supportive care for the newborn [3,10,12]. FCC is based on naturalistic observation of the infant during interaction with the caregiver to identify the strengths and vulnerabilities of the infant, caregiver, and environment. Suggestions derived from the observation include specific ways to structure the infant’s physical environment, such as reducing sounds, lights, and activity in the room, shielding the incubator with a blanket to protect the infant from surrounding light and sounds, providing a specific position that allows infants to tuck in and bring their hands close to their mouth and body, and encouraging parents to be the primary caregiver. NICU professionals are trained to recognize the requirements of preterm infants and to assist parents in fostering a strong relationship with their baby, thereby enhancing their parental competencies and self-efficacy [12,26,27].

### 3.3. Brain Magnetic Resonance Imaging (MRI) Protocol and Classification

The included infants underwent conventional brain MRI examinations at TEA during spontaneous sleep. The MRI was performed using a 1.5-T scanner (Intera; Philips Medical Systems, Best, The Netherlands). Conventional images were obtained using T1-weighted (SE 600/20), T2-weighted (FSE 6000/150), and inversion recovery (IR 3500/15/950). MRI images were independently assessed for abnormal signal intensities by two experienced observers blinded to perinatal data and outcomes. In case of disagreement, the images were re-examined by two observers together to find a consensus. A consensus was reached in all cases.

Brain MRI abnormalities were classified according to the previously published criteria [19], as follows:

- Germinal Matrix Hemorrhage–Intraventricular Hemorrhage (GMH-IVH): The germinal matrix is a structure that is normally visible on imaging and undergoes involution as the fetus ages, leaving behind only remnants in the caudothalamic notch and roof of the temporal horns after 32 weeks of gestation. In our study, an irregular contour in a subependymal area with a low T2 signal or accompanying IVH was categorized as GMH-IVH. It is important to note that hemorrhage may not be immediately evident at TEA.
- Hemorrhagic Parenchymal Infarction (HPI): When fully developed, HPI is characterized by a focal bulging or outpouching of the ventricular contour, usually unilateral. This is often accompanied by a low T2 signal component, indicating previous hemorrhage.
- Periventricular Leukomalacia (PVL): PVL typically manifests as multiple bilateral periventricular cysts in a symmetric distribution, initially appearing separate from the ventricle. Solitary or unilateral cysts are more likely to be venous infarcts or connatal cysts. At TEA, the cysts may no longer be visible. Our study used the following criteria to diagnose PVL: residual bilateral periventricular cysts, dilated/angulated posterior aspects of the lateral ventricles, and associated white matter volume loss. This is often accompanied by thalamic atrophy and abnormal myelination.
- Punctate White Matter Lesions (PWML): PWML are small areas of injury in the white matter with no consistent sonographic appearance. In our study, PWML are defined as small foci of high T1 signal in the white matter, less commonly seen on T2 sequences. The conspicuity of these lesions decreases over time, suggesting a likely higher original lesion load.
- Subependymal Cysts: Subependymal cysts, characterized by thin walls, are typically located at the caudothalamic notch. These cysts may or may not be the sequelae of a germinal matrix hemorrhage.
- Cerebellar Hemorrhage: Cerebellar hemorrhage is defined by size, with small hemorrhages smaller than 5 mm and large hemorrhages larger than 5 mm. At TEA, cerebellar atrophy may be present with minimal evidence of hemorrhage. It is noteworthy to mention that the germinal matrix is located on the outer surface of the cerebellum, so hemorrhages may involve the cerebellar cortex.
- Abnormal Myelin: Abnormal myelin is defined as a diffuse excessively high T2 signal intensity in the subcortical white matter.
- Unclassified Lesions: This category included findings that did not conform to the lesion definitions above. Lesions were considered major if associated with lobar tissue or multiple areas of tissue loss.

### 3.4. Neurodevelopmental Follow-Up

Neurodevelopmental follow-up was performed by a multidisciplinary team comprising an experienced neonatologist in neuro-developmental neurology, a child psychologist, and a physiotherapist, as previously detailed [6,9]. To ensure adherence, parents received timely telephone reminders for appointments. Surviving infants underwent assessments using the Amiel-Tison neurological assessment [28] and the Griffiths Mental Developmental Scales (GMDS-R) [29]. The GMDS-R (0–2 years) yields a global development quotient (DQ) for infants’ abilities and five subscale quotients (locomotor, eye and hand coordination, personal and social, hearing and language, cognitive performance), each with a mean of 100 and a standard deviation (SD) of 15. The GMDS-R abnormality cutoff was two SDs below the normative mean [29]. The GMDS-R results were compared among the groups of different GAs. Neurodevelopmental outcomes were classified as major sequelae or severe outcome (cerebral palsy, DQ ≤ 70, severe sensory impairment), minor sequelae (minor neurological signs such as clumsiness or DQ between 71 and 85), and normal outcomes (no neurological signs and DQ > 85) [9,30]. CP was classified according to the Gross Motor Function Classification System (GMFCS) [31].

## 4. Statistical Analysis

Continuous variables were reported as mean and standard deviation, while categorical variables were expressed as counts and percentages. χ^2^ test was used for categorical variables, whereas continuous variables were compared using Mann–Whitney, ANOVA, or Kruskal–Wallis tests, as appropriate. The correlation between variables was evaluated using Spearman’s coefficient. Risk factors for major sequelae were evaluated using univariate regression analysis models; significant risk factors were included in a multivariate logistic model. The results were reported as odds ratio (OR) and 95% confidence interval (IC). A *p* value < 0.05 was considered statistically significant. The statistical analysis was performed using MedCalc statistical software (version 22.007, July 2023).

## 5. Results

**Figure 1:**
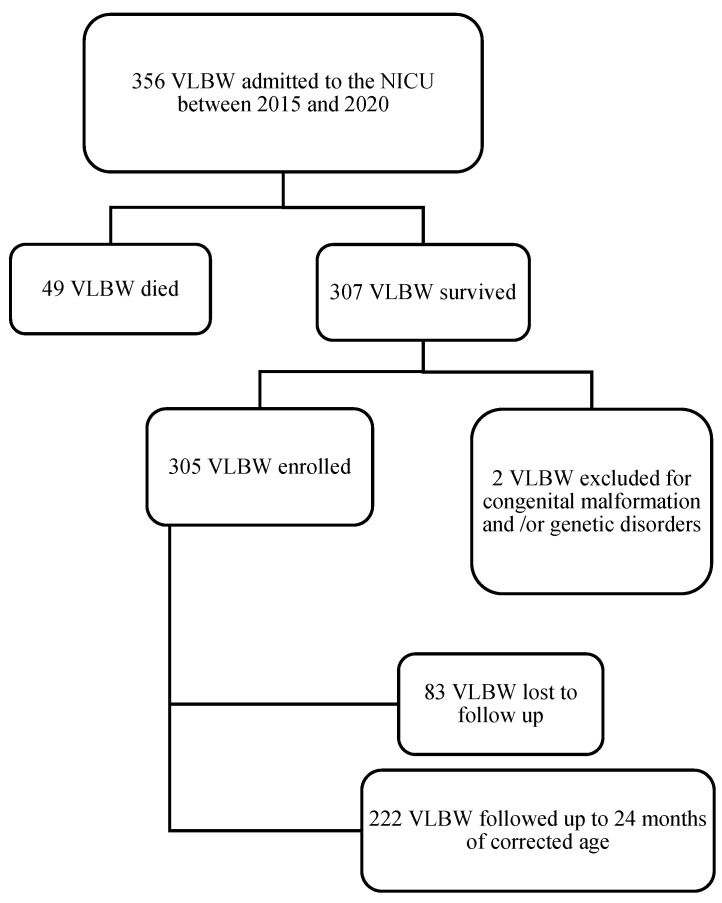
Study flow diagram.

**Figure 2:**
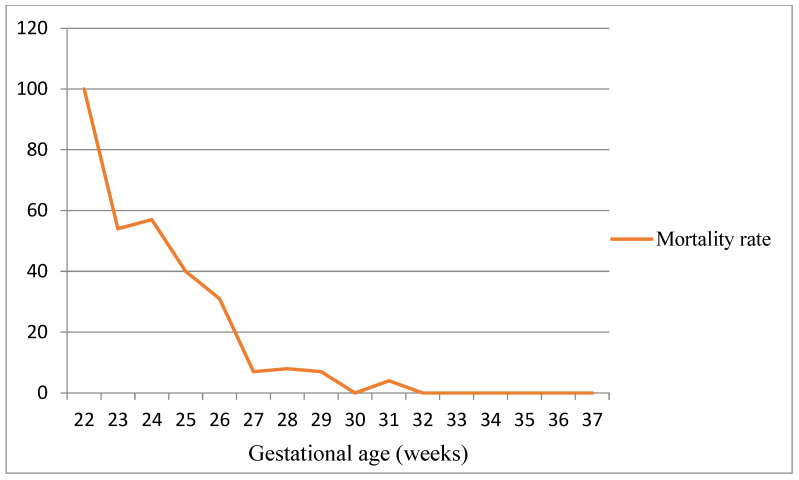
Mortality rate in relation to gestational age at birth.

Among the 356 VLBW infants admitted to the NICU of Modena, 354 were eligible for inclusion in the study (Figure 1). In total, 49 of the 356 (13.8%) infants died before hospital discharge. Figure 2 shows the mortality rate in relation to GA. GA and BW were significantly lower in the group of dead infants (mean GA = 25.4 weeks ± 2.0; mean BW = 707.2 g ± 261.7) compared to survivors (mean GA = 29.2 weeks ± 2.8; mean BW = 1131.8 g ± 277.8) (*p* < 0.001). Out of 354 (27.2%) infants, 83 were lost to follow-up. Italian infants were predominant in the group that completed the 24-month follow-up (68.8%) (*p* < 0.0001). Two hundred twenty-two infants (72.8%) completed the follow-up and were included in the study (Figure 1). In the study population, 12 out of 221 newborns were outborn (5.4%). The mean GA was comparable between the inborn and the outborn groups (mean GA = 29.0 weeks ± 2.7 in inborn and mean GA = 28.7 weeks ± 1.9 in outborn). Infants lost to follow-up exhibited GA and birth weight (BW) similar to infants who completed the follow-up (GA = 29.4 weeks ± 3.0 and BW = 1162.2 g ± 319.5; GA = 29.0 ± 2.6 and BW = 1118.3 g ± 264.5, respectively).

### 5.1. Neurodevelopmental Outcome

Neurodevelopmental outcomes at 24 months of CA were classified as normal in 173/222 children (77.9%), minor sequelae in 34 cases (15.3%), and major sequelae in 15 cases (6.8%). Among the 15 infants with major sequelae, 8 infants (3.6% of the entire study group) had CP (5 with non-disabling CP), while 6 infants (2.7% of the entire sample) had DQ < 70. Five infants had severe visual impairment, including four with concomitant CP (Table 1).

**Figure 3:**
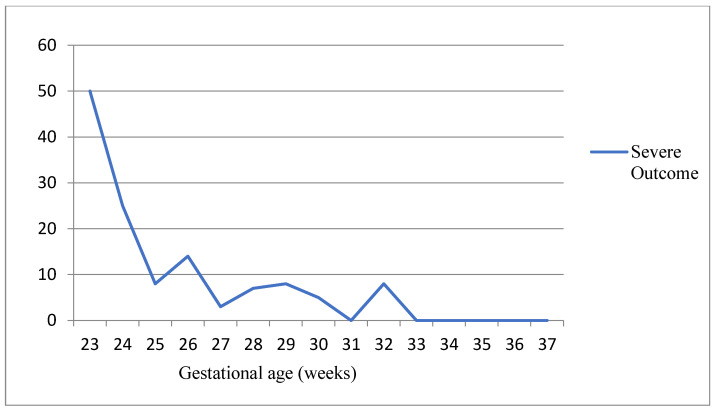
Severe neurodevelopmental outcomes in relation to gestational age at birth.

Figure 3 shows the rate of infants with neurodevelopmental outcomes in relation to GA at birth (Spearman’s coefficient = 0.257 and *p* < 0.0001). Among the 98 patients with GA ≤ 28 weeks, 9 had major (9.2%) and 21 had minor sequelae (21.4%), whereas patients with GA > 28 weeks had a lower rate of abnormal outcomes (6/124 had (4.8%) and 13/124 minor sequelae (10.5%)) (*p* = 0.0241) (Table 2). In total, 12 of the 15 (80.0%) infants with major sequelae and 26/34 (76.5%) infants with minor sequelae had a GA < 29 weeks. All children with CP had a GA < 29 weeks.

Perinatal data were compared between the groups with different neurodevelopmental outcomes (Table 3). In the univariate analysis, GA, Apgar score, mechanical ventilation, PDA, ROP > grade 2, late-onset sepsis, PIH, and breast milk at discharge were significantly associated with neurodevelopmental outcomes (Table 4). In the multivariate regression model, only ROP (OR 1.8; *p* = 0.016) and PIH (OR 5.6; *p* < 0.004) were associated with severe outcomes (Table 4).

**Figure 4:**
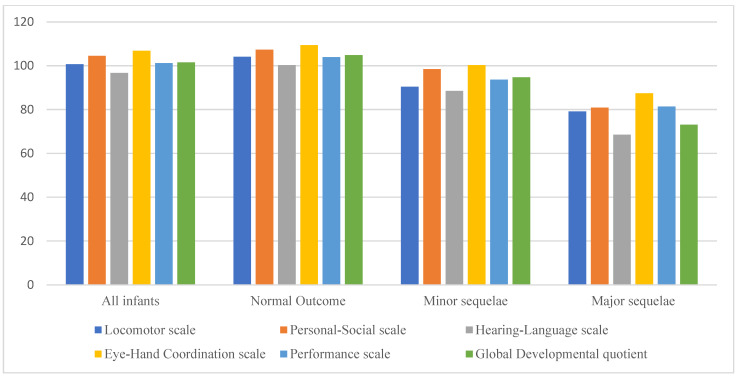
Developmental profile of the study population according to neurodevelopmental outcomes at 24 months of corrected age *. * Mean values of Griffiths Mental Development Quotient and subscales are reported. Hearing–Language scale was significantly lower than other scales in all groups (*p* < 0.01).

**Figure 5:**
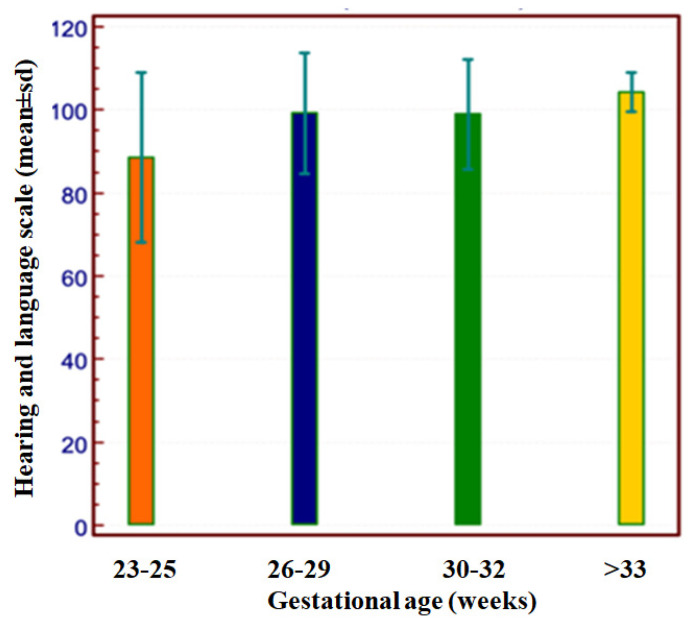
Hearing and Language Griffiths Mental Development subscale in infants without major sequelae.

The results of the GMDS DQ and the subscales are shown in Figure 4 and Supplementary Table S1. The Hearing–Language subscale was significantly lower than the other subscales in the whole study group and in all groups with different outcomes (*p* < 0.01). The GMDS DQ and subscales were compared among groups with different GA, excluding patients with severe outcomes: preterm infants born at the lowest gestational age (23–25 weeks of GA) presented significantly lower hearing and language scores than infants born at a higher gestational age (Supplementary Table S2 and Figure 5).

### 5.2. Brain MRI Abnormalities

**Figure 6:**
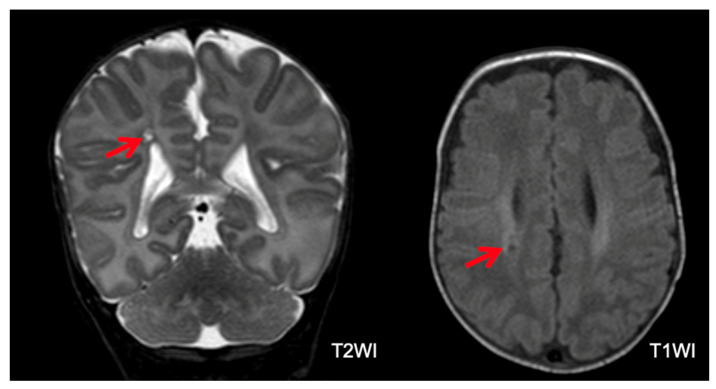
Microcystic periventricular leukomalacia in a patient with severe outcomes. White matter reduction with ventricular dilatation and microcysts near the right lateral ventricle (arrows).

**Figure 7:**
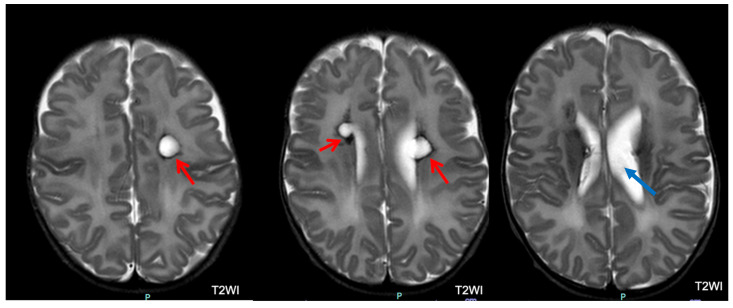
Macrocystic periventricular leukomalacia in patients with severe outcomes. White matter reduction with left ventricular dilatation (blue arrow) and bilateral periventricular macrocysts (red arrows).

**Figure 8:**
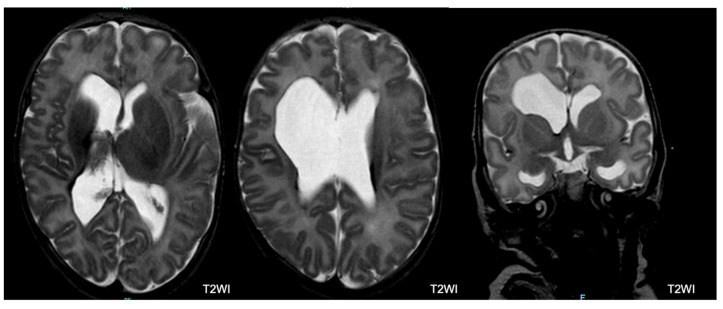
Ventricular dilatation with ex vacuo severe dilatation of the right ventricle frontal horn following intraventricular hemorrhage (IVH) and right hemorrhagic parenchymal infarction (HPI), in a patient with severe outcome.

Brain MRI was performed in 221 (99.5%) of 222 infants (one infant with normal CUS and a normal outcome did not undergo brain MRI). Among the 221 infants with brain MRI, 78 (35.3%) had normal MRI. Among the remaining 143 infants, 70 (48.9%) had an abnormal MRI with multiple lesions (Figure 6, Figure 7 and Figure 8). Major lesions (GMH-IVH, HPI, PVL, major unclassified or cerebellar hemorrhage > 5 mm) were confirmed in 76 of 221 (34.4%) infants. Several cerebral lesions (any lesion, cerebral hemorrhage > 5 mm, GMH-IVH, HPI, ventricular dilatation, PVL, and major unclassified lesions) were more common in patients with severe outcomes (Table 5). Sensitivity, specificity, positive (PPV), and negative (NPV) predictive values for each lesion are presented in Supplementary Table S3.

### 5.3. MRI and Outcome

No MRI abnormalities: Among the 78 infants with normal MRI, 68 (87.2%) had normal outcomes. Normal MRI was significantly correlated with normal outcomes (*p* < 0.001).

GMH–IVH: In total, 54 of 221 infants (24.4%) had GMH–IVH, of whom 40 (74%) had concurrent lesions. In addition, 34 of 54 (63%) children with GMH-IVH had normal outcomes.

HPI: In total, 6 of 221 (2.7%) infants had HPI, 16.7% of whom (1 = 1/6) had normal development at 24 months.

PVL: Out of 221 (2.2%) infants, 5 had PVL, of whom 3 (60.0%) had abnormal development at 24 months. The presence of PVL was highly specific for abnormal outcomes (Supplementary Table S3).

PWML: A total of 11 out of 221 (5.0%) infants had punctate white matter lesions, of whom 8 (72.7%) had additional lesions. Moreover, 9 of 11 (81.8%) infants with PWML had normal neurodevelopmental outcomes at 24 months.

Subependymal Cysts: Out of 221 (70.6%) infants, 17 had subependymal cysts, 70.6% (*n* = 12/17) of whom had additional lesions. In addition, 13 of 17 (76.5%) infants had normal outcomes.

Cerebellar Hemorrhage: Cerebellar hemorrhage occurred in 13/221 (5.9%) infants; 92.3% (*n* = 12/13) had additional lesions; 4 of 221 (1.8%) infants had a large cerebellar hemorrhage, 25.0% of whom (*n* = 1/4) had normal neurodevelopmental outcomes; 9 of 221 (4%) had a small cerebellar hemorrhage, 66.7% of whom (*n* = 6/9) had normal neurodevelopment at 24 months.

Ventriculomegaly: Ventriculomegaly was the most common type of lesion in our cohort of preterm infants (*n* = 76/221; 34.4%); 63.2% (*n* = 48/76) had additional lesions. In total, 53 of 76 (69.8%) infants developed normally at 24 months.

**Figure 9:**
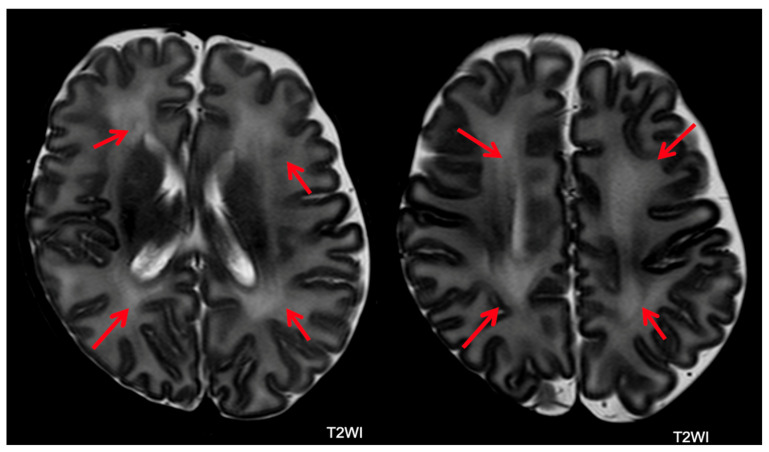
Abnormal myelin. White matter hyperintensity (arrows) in a patient with minor sequelae.

Abnormal myelin: Out of 221 (26%) infants, 57 had abnormal myelin (Figure 9), of whom 7.0% (*n* = 4/57) had concurrent lesions. Furthermore, 43 of 57 (75.4%) children had normal outcomes at 24 months.

Corpus Callosum Thinning: A total of 9 out of 221 (4%) infants had corpus callosal thinning, 55.6% of whom (*n* = 5/9) had normal development at 24 months.

Unclassified Lesions: Out of 221 (5%) infants, 11 had unclassified lesions; 100% had a concomitant lesion. Four infants had minor unclassified lesions, 75% of whom (*n* = 3/4) had normal neurodevelopment at 24 months. Among seven children with major unclassified lesions, two (28.6%) had normal outcomes.

As reported in Supplementary Table S3, the presence of any cerebral lesion on MRI showed high sensitivity and NPV. GMH-IVH and ventriculomegaly had the highest sensitivity for a severe outcome. Cerebellar hemorrhage, HPI, PWML, thin corpus callosum, PVL, and unclassified lesions showed high specificity. GMH-IVH and ventriculomegaly had the highest diagnostic accuracy.

## 6. Discussion

This study reports neurodevelopmental and neuroimaging data from a cohort of VLBW infants admitted to an Italian NICU undergoing FCC, spanning a recent 6-year period. Infants who died (13.8%) had a lower GA and BW than survivors, suggesting a potentially higher burden of brain damage in non-survivors. Severe neurodevelopmental outcomes occurred in 6.8% of survivors, with CP in 3.6% of cases. More than half of CP cases were non-disabling, and a substantial proportion showed satisfactory cognitive development. Although a few patients (2.7%) had severe mental delay (DQ < 70), moderate developmental delay was quite common (32.6% of infants with moderate to severe sequelae had a DQ below 85). Most of the largest studies on the neurodevelopmental outcomes of preterm infants date back to the 2000s and have demonstrated variable rates of severe adverse neurological outcomes. For example, the EPIPAGE study reported survival rates without neurodevelopmental impairment at 2 years of CA of 48.5%, 90.0%, and 97.5% for infants born at 22–26, 27–31, and 32–34 weeks of gestational age, respectively. The overall rates of CP were 6.9%, 4.3%, and 1.0% at 24–26, 27–31, and 32–34 weeks of gestation, respectively [32]. In a Swedish cohort of extremely preterm infants born before 27 weeks of gestation and evaluated at 30 months of corrected age, 42% had no disability, 31% had mild disability, 16% had moderate disability, and 11% had severe disability, with a CP prevalence of 7%. Moderate or severe overall disability decreased with GA at birth [33]. The Victorian Infant Collaborative Study Group evaluated neurodevelopmental outcomes at 24 months of CA in infants born at 22–27 weeks of gestation in the state of Victoria in 1991 and 2005 and reported CP rates of 11% and 9.8%, respectively [34]. A recent (2013–2018) and large study on extremely low birth weight infants in the United States showed that among infants born at less than 27 weeks’ GA, rehospitalization (49.9%) and neurodevelopmental impairment (21.2%) were common at 2 years of age [30].

Our study reveals one of the lowest incidences of severe outcomes when compared to previously published studies [3,30,32,33,34,35,36]. In our study population, CP was less frequent and less severe than previously reported, and patients with CP often showed satisfactory cognitive development. Moreover, severe global developmental delay (DQ < 70) was not as prevalent as that in previous studies. Although the reasons for these differences are uncertain, several factors may be involved, such as the small sample of infants included in the study and the underrepresentation of patients with a lower GA. Furthermore, in most of the previous studies, the time of enrollment extended back several years, potentially resulting in variations in populations and the treatments administered. Additionally, the quality of medical care and adherence to the FCC model in our NICU could partially account for the differences in unfavorable outcomes. Interestingly, in the lower GA categories, our patients exhibited a distinct disharmonic neurodevelopmental profile at 24 months of CA, which was marked by a lower language domain score in patients without major sequelae. As a matter of fact, preterm infants with lower GA (23–25 weeks) and DQ within the normal range showed significantly lower Hearing–Language scores compared to those with higher GA. Preterm infants are at a higher risk of language impairments, which can arise from an early age and persist through school age or adolescence [3,37,38,39,40,41,42]. Our findings are consistent with previous research demonstrating that language impairment, detectable as early as 24 months of CA, is not necessarily linked to cognitive disability [3,37,38,39,40,41,42]. The disharmonic profile identified in our sample may be attributed to a combination of risk factors, but it is mostly associated with severe prematurity, and we cannot exclude the possibility that these children may later develop learning and/or language disorders. Our study highlights the need to improve neuroprotective interventions for preterm neonates by optimizing their early sensory experiences in the NICU. At the time of the study, interventions involving maternal voice were applied only to selected cases [43]. However, given the results of this study, it is desirable to extend these interventions to all preterm neonates admitted to the NICU [3,43].

The multivariate analysis identified PIH and ROP as risk factors for severe neurodevelopmental outcomes at 24 months of CA. The correlation between ROP and neurodevelopmental outcomes is consistent with the current literature. A systematic review [44] indicated that infants with “any ROP” are at a higher risk of cognitive impairment or intellectual disability, CP, and behavioral problems. Additionally, brain injury is a recognized risk factor associated with adverse neurodevelopmental outcomes, and ultrasound-documented brain lesions occurred in more than 80% of patients with severe outcomes (PIH in 67% and PVL in 20%). In contrast to previous studies, cerebral damage was more accurately assessed using brain MRI. In our study, 64% of patients displayed brain MRI abnormalities. The rates of HPI, IVH, and PVL were lower than those of their PIH and PVL counterparts diagnosed by CUS. This may be because hemorrhage may not be readily apparent at TEA. Moreover, PVL may be incorrectly detected by CUS. However, the comparison between CUS and MRI was not within the scope of this study. Concerning the correlation between MRI findings and neurodevelopmental outcomes, we found that a normal brain MRI was significantly correlated with a normal neurodevelopmental outcome. Conversely, the presence of any brain MRI abnormality was associated with variable outcomes. HPI and PVL were significantly correlated with severe outcomes. However, other lesions, such as ventriculomegaly, abnormal myelin, and GMH-IVH, were correlated with variable outcomes. Possibly, the degree of these lesions played a role in determining the presence and severity of the neurodevelopmental sequelae. Interestingly, a small proportion of infants with normal brain MRI (12.8%) developed neurological sequelae, mostly cognitive impairment. This may be due to conventional MRI-undetectable cerebral abnormalities related to premature birth and extrauterine brain development, like reduced cortical fold, abnormal neural connectivity, or atypical synaptic connections [3].

The strengths of our study lie in its prospective design and a notably recent enrollment period. Indeed, the enrollment period (2015–2020) is very close to the present day, reflecting the effect of the most advanced intensive support and neuroprotective care for newborns (like FCC) on neonatal outcomes. In contrast, most previous studies included cohorts of preterm infants born years earlier, although these cohorts were larger [3,32,33,34]. Furthermore, our study is one of the first to investigate the neurodevelopmental outcomes and MRI abnormalities of VLBW infants born in Italy.

Our study has some limitations that should be noted. The first major limitation is the small sample size and the limited socio-demographic data. A second limitation is the incomplete follow-up data for 27% of the neonates, a factor that some may contend introduces a potential selection bias. We cannot exclude the fact that the COVID-19 pandemic (which occurred in Italy from 2020 to 2021) had an impact on the follow-up dropout. However, previous studies reported similar dropout rates and suggested that poorly performing children are often overrepresented among those not evaluated, particularly among children from more disadvantaged families [9,45]. In the current study, infants who completed follow-up exhibited gestational age and birth weight comparable to those who did not. Therefore, although we lack information on the socioeconomic status of the children lost to follow-up, we presume that our results do not underestimate the adverse outcomes. Nevertheless, efforts to improve follow-up compliance should be pursued to address this limitation. Finally, the follow-up was not extended beyond the age of 2 years of CA. It is well known that evaluations within the first two years of a child’s life primarily identify major disabilities, whereas identifying minor anomalies (motor, cognitive, and behavioral) is challenging in early life and requires extended evaluations until school age. Recent data showed a lower prevalence of severe motor outcomes such as CP and severe cognitive deficits in preterm children. There is an increasing trend in neurodevelopmental disorders (behavioral, social competence, learning, and executive function disorders) that manifest later in childhood among preterm children, and therefore require long-term follow-up, at least until 6–7 years of age [3,32,33,34,35,36,37,38,39,40,41,42]. Nevertheless, follow-up during the first 2 years of life is essential to identify mental, neuromotor, and neurosensory impairments. Neurodevelopmental follow-up within the initial 2 years of life stands out as a highly sensitive tool for the early detection of developmental delays, enabling the referral of high-risk children to early intervention. Addressing these deficits during the first years of life is crucial because it is a very sensitive period for brain development, with the highest expression of brain plasticity [46].

In conclusion, our study showed a low rate of severe neurodevelopmental outcomes in a population of VLBW infants born in Italy between 2015 and 2020 who were undergoing FCC. In the lower GA categories, we found a distinct disharmonic neurodevelopmental profile with a significant language acquisition delay. PIH and ROP were strongly associated with severe neurodevelopmental outcomes. Furthermore, a normal brain MRI was significantly correlated with normal outcomes. Our study supports the need for further follow-up programs to establish a national network perspective and extend the age of assessment. These networks will play a crucial role in promoting access to formal neurodevelopmental assessments and facilitating timely rehabilitative interventions.

## Figures and Tables

**Table 1:** Neurodevelopmental outcomes of the study population at 24 months of corrected age.

Neurodevelopmental Outcome	n (%)
**Normal**	173 (77.9)
**Minor sequelae**	34 (15.3)
DQ between 76 and 87	10 (4.5) ^a^
Clumsiness	19 (8.6)
**Major sequelae**	15 (6.8)
Disabling CP	3 (1.4)
Non-disabling CP	5 (2.3)
Severe visual impairment (peripheral or central ^b^)	5 (2.3) ^c^
DQ < 70 without other impairments	6 (2.7)

DQ, global developmental quotient; CP, cerebral palsy. ^a^ 3 infants had clumsiness; ^b^ 3 children had peripheral and 2 had central severe visual impairment; ^c^ 4 children had CP. CP is defined as disabling when GMFC > 2.

**Table 2:** Neurodevelopmental outcomes and cerebral palsy in relation to gestational age at birth.

	GA 22–25 Weeks	GA 26–29 Weeks	GA 30–33 Weeks	GA > 33 Weeks	*p* Value
	*n* = 19	*n* = 114	*n* = 80	*n* = 9	
Normal, *n* (%)	11 (57.9)	84 (73.7)	69 (86.3)	9 (100.0)	0.067
Minor sequelae, *n* (%)	5 (26.3)	21 (18.4)	8 (10.0)	0 (0.0)	
Major sequelae, *n* (%)	3 (15.8)	9 (7.9)	3 (3.8)	0 (0.0)	
CP, *n* (%)	1 (5.3)	6 (5.3)	0 (0.0)	0 (0.0)	0.188
No CP, *n* (%)	18 (94.7)	108 (94.7)	80 (100.0)	9 (100.0)	

GA: gestational age. CP: cerebral palsy.

**Table 3:** Perinatal clinical data according to neurodevelopmental outcomes.

	All Infants	Normal Outcome	Minor Sequelae	Major Sequelae	*p* Value
	*n* = 222	*n* = 173	*n* = 34	*n* = 15	
Maternal age, mean (SD), years	34.3 (5.4)	34.6 (5.4)	33.7 (6.1)	30.7 (3.2)	0.082
Multiple gestations, *n* (%)	69.0 (31.1)	56.0 (32.4)	10.0 (29.4)	3.0 (20.0)	0.595
Cesarian section, *n* (%)	90 (40.6)	71 (41.0)	10 (29.4)	9 (60.0)	0.126
Antenatal steroid therapy, *n* (%)	202 (91.0)	158 (91.3)	31 (91.2)	13 (86.7)	0.788
Magnesium sulphate, *n* (%)	166 (74.8)	123 (71.1)	29 (85.3)	14 (93.3)	0.051
Gestational age, mean (SD), weeks	29.0 (2.6)	29.3 (2.6)	27.8 (2.3)	27.6 (2.7)	<0.001
Male, *n* (%)	115 (51.8)	87 (50.3)	20 (58.8)	8 (53.3)	0.656
Ethnicity, *n* (%)	71 (32.0)	44 (25.4)	21 (61.8)	6 (40.0)	0.0001
Birth weight, mean (SD), g	1118.3 (264.5)	1154.8 (248.4)	984.8 (265.6)	1000.1 (324.2)	0.001
AP1, mean (SD)	5.8 (2.11)	6.0 (2.1)	5.7 (2.0)	4.3 (2.1)	0.009
AP5, mean (SD)	7.8 (1.6)	8.0 (1.6)	7.8 (1.5)	6.9 (2.3)	0.086
Cardiac compressions, *n* (%)	14 (6.3)	10 (0.6)	2 (5.9)	2 (13.3)	0.515
Resuscitation, *n* (%)	74 (33.3)	50 (28.9)	16 (47.1)	8 (53.3)	0.054
Epinephrin, *n* (%)	9 (4.1)	6 (3.5)	1 (2.9)	2 (13.3)	0.169
Intubation at birth, *n* (%)	76 (34.2)	51 (29.5)	17 (50.0)	8 (53.3)	0.021
Mechanical Ventilation, *n* (%)	87 (39.2)	57 (32.9)	19 (55.9)	11 (73.3)	0.0009
nCPAP, *n* (%)	167 (75.2)	132 (76.3)	24 (70.6)	11 (73.3)	0.731
Surfactant, *n* (%)	179 (80.6)	84 (48.6)	22 (64.7)	11 (73.3)	0.068
Oxygen 28 days, *n* (%)	102 (46.0)	68.0 (39.3)	24.0 (70.6)	10 (66.7)	0.002
Bronchopulmonary dysplasia, *n* (%)	48 (21.6)	28 (16.2)	14 (41.2)	6 (40.0)	0.001
PDA, *n* (%)	79 (35.6)	54 (31.2)	16 (47.1)	9 (60.0)	0.026
PDA treated pharmacologically, *n* (%)	52 (23.4)	33 (19.1)	15 (44.1)	4 (26.7)	0.007
NEC, *n* (%)	6 (2.7)	4 (2.3)	1 (2.9)	1(6.7)	0.605
Early-onset sepsis, *n* (%)	2 (0.9)	1 (0.6)	1 (2.9)	0 (0)	0.382
Late-onset sepsis, *n* (%)	35 (15.8)	18 (10.4)	10 (2.9)	7 (46.7)	0.0001
ROP > grade 2, *n* (%)	10 (4.5)	6 (3.5)	1 (2.9)	3 (20.0)	0.0004
ROP surgery, *n* (%)	13 (5.9)	8 (4.6)	2 (5.9)	3 (20.0)	0.052
PIH, *n* (%)	51 (23.0)	33 (19.1)	8 (23.5)	10 (66.7)	0.0001
PVL >1, *n* (%)	7 (3.2)	3 (1.7)	1 (2.9)	3 (20.0)	0.0005
Human milk discharge, *n* (%)	154 (69.4)	125 (72.3)	23 (67.6)	6 (40.0)	0.033

AP1: Apgar at the 1st minute. AP5: Apgar at the 5th minute. nCPAP: nasal continuous positive airway pressure. PDA: Patent Ductus Arteriosus. NEC: necrotizing enterocolitis. ROP: retinopathy of prematurity. PIH: periventricular–intraventricular hemorrhage. PVL: periventricular leukomalacia. Bronchopulmonary dysplasia: oxygen supplementation at 36 weeks of post-conceptional age.

**Table 4:** Univariate and multivariate analysis of risk factors for severe neurodevelopmental outcomes.

	Univariate Analysis			Multivariate Analysis		
	OR	CI 95%	*p* Value	OR	CI 95%	*p* Value
Maternal age	0.9	0.8–1.0	0.105			
Maternal education	3.6	0.4–30.4	0.235			
Multiple gestations	0.5	0.1–2.0	0.319			
Cesarean section	2.3	0.8–6.7	0.123			
Antenatal steroid therapy	0.6	0.1–2.8	0.525			
Magnesium sulphate	5.1	0.7–39.4	0.053			
Gestational age	0.8	0.6–1.0	0.027			
Male	1.1	0.4–3.1	0.902			
Ethnicity	1.5	0.5–4.3	0.498			
Birth weight	1.0	1.0–1.0	0.075			
AP1	0.7	0.6–0.9	0.006			
AP5	0.7	0.6–1.0	0.027			
Cardiac compressions in delivery room	2.5	0.5–12.3	0.305			
Any resuscitation in delivery room	5.3	0.6–44.1	0.062			
Epinephrin in delivery room	4.4	0.8–23.2	0.125			
Intubation in delivery room	2.3	0.8–6.7	0.119			
Mechanical Ventilation	4.7	1.4–15.3	0.006			
nCPAP	0.9	0.3–2.9	0.836			
Surfactant	2.6	0.8–8.3	0.098			
Oxygen 28 days	2.7	0.8–9.1	0.081			
Bronchopulmonary dysplasia 36 w	2.7	0.9–8.2	0.091			
PDA	2.9	1.0–8.6	0.046			
PDA treated pharmacologically	1.2	0.4–4.0	0.762			
NEC	2.9	0.3–26.4	0.400			
Early-onset sepsis	0.0	0.0–0.0	0.596			
Late-onset sepsis	5.6	1.9–16.6	0.003			
ROP > grade 2	2.0	1.3–3.0	0.004	1.8	1.1–2.8	0.016
ROP surgery	4.9	1.2–20.3	0.048			
PIH	8.1	2.6–25.0	<0.001	5.6	1.7–18.4	0.004
Human milk discharge	0.3	0.1–0.8	0.015			
Outborn	3.0	0.6–15.0	0.170			

The univariate and multivariate analysis for cystic PVL was not solvable.

**Table 5:** Cerebral MRI lesions and neurodevelopmental outcomes.

	All Infants	Normal Outcome	Minor Sequelae	Major Sequelae	*p* Value
	*n* = 221	*n* = 173	*n* = 34	*n* = 15	
Any lesion	143 (64.4)	105 (60.7)	24 (70.6)	14 (93.3)	0.032
Abnormal myelin	56 (25.2)	43 (25.9)	8 (23.5)	5 (33.3)	0.750
Cerebellar hemorrhage >5 mm	4 (1.8)	1 (0.6)	0 (0)	3 (20.0)	<0.0001
Cerebellar hemorrhage < 5 mm	9 (4.1)	6 (3.5)	1 (2.9)	2 (13.3)	0.169
GMH-IVH	54 (24.3)	34 (19.7)	10 (29.4)	10 (66.7)	0.0002
HPI	6 (2.7)	1 (0.6)	2 (5.9)	3 (20.0)	<0.0001
White matter punctate lesions	11 (5.0)	9 (5.2)	1 (2.9)	1 (6.7)	0.814
Subependymal cyst	17 (7.7)	13 (7.5)	3 (8.8)	1 (6.7)	0.957
Thin corpus callosum	9 (4.1)	5 (2.9)	3 (8.8)	1 (6.7)	0.244
Ventricular dilatation	76 (34.2)	53 (30.6)	13 (38.2)	10 (66.7)	0.017
PVL	5 (2.3)	2 (1.2)	1 (2.9)	2 (13.3)	0.009
Major unclassified	7 (3.2)	2 (1.2)	1 (2.9)	4 (26.7)	<0.0001
Minor unclassified	4 (1.8)	3 (1.7)	0 (0)	1 (6.7)	0.248

GMH-IVH: Germinal Matrix Hemorrhage–Intraventricular Hemorrhage. HPI: hemorrhagic parenchymal infarction. PVL: periventricular leukomalacia.

## Data Availability

The data presented in this study are available on request from the corresponding author. The data are not publicly available due to privacy.

## Supplementary Materials

The following supporting information can be downloaded at: https://www.mdpi.com/article/10.3390/children11010012/s1, Table S1. Griffiths Mental Development Quotient and Subscale among patients with different neurodevelopmental outcome. Table S2. Griffiths Mental Development quotient and subscales according to gestational age. Table S3. Cerebral MRI abnormalities sensitivity, specificity, positive and negative predictive values, and area under the receiver operating characteristic curve for severe outcome.
