# Supplementary material for: Neurodevelopmental Outcome and Neuroimaging of Very Low Birth Weight Infants from an Italian NICU Adopting the Family-Centered Care Model
Source: Children (Basel). 2023 Dec 21;11(1):12. doi: 10.3390/children11010012 (PMC10813860; doi:10.3390/children11010012)
Supplement: Supplementary file 1 [file children-11-00012-s001.zip › children-2752476-supplementary.pdf]

Supplementary Table S1. Griffiths Mental Development Quotient and Subscale among patients with different neurodevelopmental outcome.

	**All infants** N=222 (mean±sd)	**Normal Outcome** N=173 (mean±sd)	**Minor sequelae** N=34 (mean±sd)	**Major sequelae** N=15 (mean±sd)
**Locomotor scale**	100.7±12.9	104.1±8.6	90.4±14.1	79.2±22.6
**Personal-Social scale**	104.5±14.3	107.3±10.7	98.4±12.9	80.9±28.4
**Hearing-Language scale ***	96.7±17.2	100.2±12.4	88.6±21.7	68.5±26.8
**Eye-Hand Coordination scale**	106.8±14.3	109.4±8.8	100.2±13.3	87.4±34.3
**Performance scale**	101.1±14.6	103.9±9.4	93.7±13.4	81.4±33.3
**Global developmental quotient**	101.5±12.2	104.8±8.0	94.7±10.5	73.1±20.1

* Hearing-Language scale resulted lower than other scales among all groups ($p<0.01$).

Supplementary Table S2. Griffiths Mental Development quotient and subscales according to gestational age.

	**GA 22-25 weeks N=16** (mean±sd)	**GA 26-29 weeks N=105** (mean±sd)	**GA 30-33 week N=77** (mean±sd)	**GA >33 weeks N=9** (mean±sd)	**All infants * N=207** (mean±sd)	***p* value**
**Locomotor scale**	97.8±6.1	101.6±9.7	103.1±8.1	103.4±4.1	100.7±12.9	0.332
**Personal-Social scale**	103.4±10.3	104.7±11.3	108.0±10.1	108.5±8.9	104.5±14.3	0.179
**Hearing-Languagescale**	88.5±28.2	99.2±18.2	99.0±16.5	104.2±5.0	96.7±17.2	**0.027**
**Eye-Hand Coordination scale**	107.2±12.6	108.2±9.6	107.3±7.2	111.3±6.7	106.8±14.3	0.695
**Performance scale**	101.8±11.6	102.3±11.2	102.1±6.8	105.7±2.8	101.1±14.6	0.817
**Global developmental quotient**	99.4±8.9	103.3±8.9	103.5±7.1	107.1±1.1	101.5±12.2	0.212

GA: gestational age. * Infants with severe outcome are excluded.

Supplementary Table S3. Cerebral MRI abnormalities sensitivity, specificity, positive and negative predictive values, and area under the receiver operating characteristic curve for severe outcome.

**Cerebral MRI abnormalities**	**Abnormal Outcome**				
	**Sensitivity (%)**	**Specificity (%)**	**PPV (%)**	**NPV (%)**	**AUC**
Any lesion	93	37	10	99	0.7
Abnormal myelin	33	75	9	94	0.5
Cerebellar hemorrhage >5 mm	20	100	75	94	0.6
Cerebellar hemorrhage < 5 mm	13	97	22	94	0.6
GMH-IVH	67	79	19	97	0.7
HPI	20	99	50	94	0.6

White matter punctate lesions	7	95	9	93	0.5
Subependymal cyst	93	8	7	94	0.5
Thin corpus callosum	7	96	11	93	0.5
Ventricular dilatation	67	68	13	97	0.7
PVL	13	99	40	94	0.6
Major unclassified	27	99	57	95	0.6
Minor unclassified	7	98	17	94	0.5

PPV: positive predictive value; NPV: negative predictive value; AUC: area under the receiver operating characteristic (ROC) curve. GMH-IVH: Germinal Matrix Hemorrhage–Intraventricular Hemorrhage. HPI: Hemorrhagic Parenchymal Infarction Periventricular Leukomalacia: PVL.
